# Supplementary material for: Beneficial effect on the soil microenvironment of Trichoderma applied after fumigation for cucumber production
Source: PLoS One. 2022 Aug 2;17(8):e0266347. doi: 10.1371/journal.pone.0266347 (PMC9345367; doi:10.1371/journal.pone.0266347)
Supplement: S1 Table — CK = Unfumigated soil after use in the laboratory; Field = Unfumigated field soil; DP = Soil fumigated with dimethyl disulfide and chloropicrin after it was used for growing seedlings in the laboratory. (DOCX) [file pone.0266347.s001.docx]

**S1_Table.** **Main physicochemical characteristics of laboratory and field soils**

| Soil treatment | NH4+-N  (mg/kg) | NO3--N  (mg/kg) | Available phosphorus(mg/kg) | Available potassium(mg/kg) | Organic matter  (g/kg) | pH  (1:2.5) | Electrical conductivity  (μs/cm) |
| --- | --- | --- | --- | --- | --- | --- | --- |
| CK | 0.48 | 609.54 | 334.68 | 994 | 29.83 | 7.36 | 882 |
| Field | 0.54 | 737.84 | 373.91 | 993 | 34.62 | 7.3 | 970 |
| DP | 2.44 | 473.23 | 439.84 | 1098 | 38.54 | 7.21 | 983 |
