# Supplementary material for: Beneficial effect on the soil microenvironment of Trichoderma applied after fumigation for cucumber production
Source: PLoS One. 2022 Aug 2;17(8):e0266347. doi: 10.1371/journal.pone.0266347 (PMC9345367; doi:10.1371/journal.pone.0266347)
Supplement: S2 Table — (DOCX) [file pone.0266347.s002.docx]

**S2_Table.** **The primers and thermal programs used for gene detection in this study**

| Target organism | Primers | Sequences (5'-3') | Thermal programs |
| --- | --- | --- | --- |
| All *Trichoderma* | M1 | CTGGCATCGATGAAGAACG | 3 min at 95°C, followed by 35 cycles of 30 s at 95°C, 30 s at 57°C, and 30 s at 72°C; 8 min at 72°C,Melt Curve 65.0 to 95.0，increment 0.5℃，for 5 s. |
|  | M2 | ATGCGAGTGTGCAAACTACTG |  |
