# Supplementary material for: Beneficial effect on the soil microenvironment of Trichoderma applied after fumigation for cucumber production
Source: PLoS One. 2022 Aug 2;17(8):e0266347. doi: 10.1371/journal.pone.0266347 (PMC9345367; doi:10.1371/journal.pone.0266347)
Supplement: S3 Table — DP30, DP265 or DP267 = Trichoderma strain 30, 265 or 267 added after fumigation (see 2.2.2. in the text for detail); DPHZ = Commercial T. harzianum added after fumigation. DP = Fumigation without Trichoderma. CK30, CK265 or CK 267 = Trichoderma strains 30, 265 or 267 added individually to soil without fumigation. CKHZ = Commercial T. harzianum added to soil without fumigation. CK = Untreated control. Means (N = 3) within the same time period accompanied by the same letter were not statistically different (P = 0.05) according to Duncan’s new Multiple-Range test. ‡ Average cfu g−1 soil of Fusarium spp. § Average cfu g−1 soil of Phytophthora spp. (DOCX) [file pone.0266347.s003.docx]

**S3_Table. Effect of fumigation combined with *Trichoderma* on the percentage reduction of soil-borne pathogens**

| Treatment | *Fusarium* spp. (%) | *Phytophthora* spp. (%) |
| --- | --- | --- |
| DP30 | 98.30a | 96.49bc |
| DP265 | 98.07a | 96.67bc |
| DP267 | 98.91a | 97.73bc |
| DPHZ | 98.91a | 98.12a |
| DP | 99.39a | 94.80bc |
| CK30 | 99.25a | 96.13bc |
| CK265 | 98.64a | 94.68c |
| CK267 | 92.89a | 97.07bc |
| CKHZ | 98.01a | 97.19bc |
| CK | (29440) ‡ | (22040) § |
