# Supplementary material for: Beneficial effect on the soil microenvironment of Trichoderma applied after fumigation for cucumber production
Source: PLoS One. 2022 Aug 2;17(8):e0266347. doi: 10.1371/journal.pone.0266347 (PMC9345367; doi:10.1371/journal.pone.0266347)
Supplement: S4 Table — DP30, DP265 or DP267 = Trichoderma strain 30, 265 or 267 added after fumigation (see 2.2.2. in the text for detail); DPHZ = Commercial T. harzianum added after fumigation. DP = Fumigation without Trichoderma. CK30, CK265 or CK 267 = Trichoderma strains 30, 265 or 267 added individually to soil without fumigation. CKHZ = Commercial T. harzianum added to soil without fumigation. CK = Untreated control. Means (N = 3) within the same time period accompanied by the same letter were not statistically different (P = 0.05) according to Duncan’s new Multiple-Range test. (DOCX) [file pone.0266347.s004.docx]

**S4_Table Changes of soil enzyme activity following different soil treatments after fumigation**

| Treatment | soil urease activity  (U/g soil) | soil sucrase activity  (U/g soil) |
| --- | --- | --- |
| DP30 | 140.26±4.5bc | 28.06±1.62c |
| DP265 | 146.75±22.32bc | 29.33±2.28c |
| DP267 | 161.74±0.33b | 29.94±2.89c |
| DPHZ | 126.93±4.16bc | 20.38±1.15d |
| DP | 111.27±14.16c | 13.96±0.92e |
| CK30 | 221.04±15.99a | 57.95±4.39a |
| CK265 | 215.21±21.82a | 44.91±0.23b |
| CK267 | 234.54±3.83a | 46.06±0.85b |
| CKHZ | 226.37±1.33a | 42.43±1.04b |
| CK | 209.55±1.17a | 41.56±1.8b |
