# Supplementary material for: Beneficial effect on the soil microenvironment of Trichoderma applied after fumigation for cucumber production
Source: PLoS One. 2022 Aug 2;17(8):e0266347. doi: 10.1371/journal.pone.0266347 (PMC9345367; doi:10.1371/journal.pone.0266347)
Supplement: S5 Table — DP267 = Trichoderma strain 267 added after fumigation (see 2.2.2. in the text for detail); DPHZ = Commercial T. harzianum added to soil after fumigation. CK267 = Trichoderma strain 267 added to soil without fumigation. CKHZ = Commercial T. harzianum added to soil without fumigation. DP = Fumigation without Trichoderma. CK = Untreated control. Means (N = 3) within the same time period accompanied by the same letter were not statistically different (P = 0.05), according to Duncan’s new Multiple-Range test. (DOCX) [file pone.0266347.s005.docx]

**S5_Table Changes in bacterial taxonomic diversity**

| Treatment | Shannon | Simpson | ACE | Chao1 |
| --- | --- | --- | --- | --- |
| DP267 | 6.0583±0.04986c | 0.0064±0.00042a | 2650.6478±25.16468c | 2627.045±35.62321c |
| DPHZ | 6.0843±0.03183c | 0.0067±0.00038a | 2684.3412±55.14374c | 2647.7557±60.06822c |
| DP | 6.0823±0.0393c | 0.0063±0.0003a | 2680.5491±31.28246c | 2685.6894±32.69162c |
| CK267 | 6.5879±0.03379a | 0.0041±0.00024b | 3731.3009±40.70377a | 3727.9614±48.04231a |
| CKHZ | 6.4935±0.0331ab | 0.0042±0.00016b | 3555.2719±63.96586b | 3518.198±66.31147b |
| CK | 6.4226±0.04721b | 0.0049±0.0003b | 3461.9901±54.29144b | 3403.7224±45.57471b |
