# Supplementary material for: Beneficial effect on the soil microenvironment of Trichoderma applied after fumigation for cucumber production
Source: PLoS One. 2022 Aug 2;17(8):e0266347. doi: 10.1371/journal.pone.0266347 (PMC9345367; doi:10.1371/journal.pone.0266347)
Supplement: S6 Table — DP267 = Trichoderma strain 267 added after fumigation (see 2.2.2. in the text for detail); DPHZ = Commercial T. harzianum added to soil after fumigation. CK267 = Trichoderma strain 267 added to soil without fumigation. CKHZ = Commercial T. harzianum added to soil without fumigation. DP = Fumigation without Trichoderma. CK = Untreated control. Means (N = 3) within the same time period accompanied by the same letter were not statistically different (P = 0.05), according to Duncan’s new Multiple-Range test. (DOCX) [file pone.0266347.s006.docx]

**S6_Table Changes in fungal taxonomic diversity**

| Treatment | Shannon | Simpson | ACE | Chao1 |
| --- | --- | --- | --- | --- |
| DP267 | 1.857±0.10331c | 0.4108±0.02212a | 207.8573±5.6627c | 212.9317±4.22215c |
| DPHZ | 3.5834±0.06105a | 0.0658±0.00574c | 246.433±16.38975bc | 250.1556±12.85672abc |
| DP | 3.3331±0.05639a | 0.079±0.01047c | 217.6043±19.8234c | 217.6815±19.38645c |
| CK267 | 2.3734±0.12209bc | 0.2356±0.01562b | 237.2058±17.50844bc | 238.4593±18.32328bc |
| CKHZ | 2.3655±0.21243bc | 0.2369±0.03819b | 276.8123±8.15185ab | 278.6448±6.08944ab |
| CK | 3.0023±0.48087ab | 0.1586±0.10562bc | 300.9261±20.47461a | 297.4378±20.91181a |
