# Supplementary material for: Beneficial effect on the soil microenvironment of Trichoderma applied after fumigation for cucumber production
Source: PLoS One. 2022 Aug 2;17(8):e0266347. doi: 10.1371/journal.pone.0266347 (PMC9345367; doi:10.1371/journal.pone.0266347)
Supplement: S7 Table — DP267 = Trichoderma strain 267 added after fumigation (see 2.2.2. in the text for detail); DPHZ = Commercial T. harzianum added to soil after fumigation. DP = Fumigation without Trichoderma CK = Untreated control. Means (N = 3) within the same time period accompanied by the same letter were not statistically different (P = 0.05), according to Duncan’s new Multiple-Range test. ‡ Average cfu g−1 soil of Fusarium spp. § Average cfu g−1 soil of Phytophthora spp. ǂ Average number of root-knot nematode (Meloidogyne spp.) per 100 g soil. (DOCX) [file pone.0266347.s007.docx]

**S7_Table Reduction of the number of soil-borne pathogens and root-knot nematodes in the field**

| Treatment | Trial 1 | | | Trial 2 | | |
| --- | --- | --- | --- | --- | --- | --- |
|  | *Fusarium* spp. | *Phytophthora* spp. | *Meloido-gyne* spp*.* | *Fusarium* spp. | *Phytophthora* spp. | *Meloido-gyne* spp. |
| DP267 | 94.18a | 83.53a | 96.81a | 81.65ab | 95.78a | 96.67a |
| DPHZ | 98.57a | 81.35a | 92.55ab | 84.25a | 93.40a | 92.55a |
| DP | 86.24b | 56.05b | 88.29c | 65.60c | 80.48b | 90.00a |
| CK | (11680)‡ | (7327)§ | (313) ^ǂ^ | (6647)‡ | (2527)§ | (100) ^ǂ^ |
