# Supplementary material for: Beneficial effect on the soil microenvironment of Trichoderma applied after fumigation for cucumber production
Source: PLoS One. 2022 Aug 2;17(8):e0266347. doi: 10.1371/journal.pone.0266347 (PMC9345367; doi:10.1371/journal.pone.0266347)
Supplement: S8 Table — DP267 = Trichoderma strain 267 added after fumigation; DPHZ = Commercial T. harzianum added to soil after fumigation. DP = Fumigation without Trichoderma. CK = Untreated control. Means (N = 3) within the same time period accompanied by the same letter were not statistically different (P = 0.05), according to Duncan’s new Multiple-Range test. (DOCX) [file pone.0266347.s008.docx]

**S8_Table The total marketable yield of cucumber.**

| Treatment | Trial 1(kg/m^2^) | | Trial 2 (kg/m^2^) | |
| --- | --- | --- | --- | --- |
|  | Mean + SE | % | Mean + SE | % |
| DP267 | 7.39±0.01a | 35.60 | 7.07±0.1a | 20.65 |
| DPHZ | 7.23±0.11ab | 32.66 | 7.04±0.05a | 20.14 |
| DP | 6.81±0.23b | 24.95 | 6.51±0.08ab | 16.21 |
| CK | 5.45±0.02c | - | 5.86±0.39b | - |
