# Supplementary figures and images for: Beneficial effect on the soil microenvironment of Trichoderma applied after fumigation for cucumber production
Source: PLoS One. 2022 Aug 2;17(8):e0266347. doi: 10.1371/journal.pone.0266347 (PMC9345367; doi:10.1371/journal.pone.0266347)

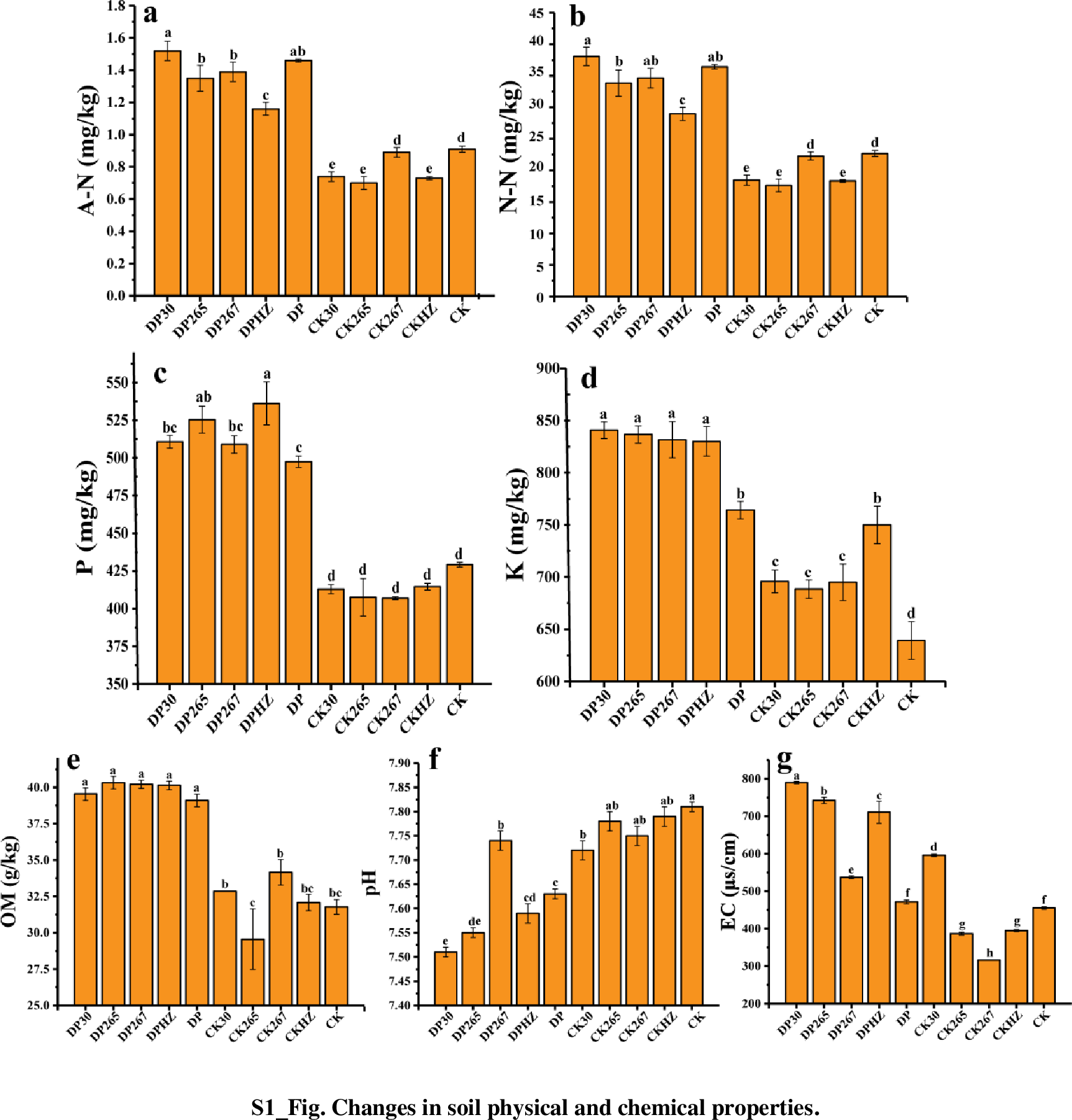

Supplement: S1 Fig — Soil Nitrogen (A-N, graph a; and N-N, graph b), Phosphorous (P, graph c), Potassium (K, graph d), Organic matter (OM; graph e), pH (graph f)), and Soil electrical conductivity (EC; graph g). DP30, DP265 or DP267 = Trichoderma spp. strain 30, 265 or 267 added after fumigation (see 2.2.2. in the text for detail); DPHZ = Commercial T. harzianum added to soil after fumigation. DP = Fumigation without Trichoderma. CK30, CK265 or CK267 = Trichoderma spp. strains 30, 265 or 267 added individually to soil without fumigation. CKHZ = Commercial T. harzianum added to soil without fumigation. CK = Untreated control. Means (N = 3) within the same time period accompanied by the same letter were not statistically different (P = 0.05), according to Duncan’s new Multiple-Range test. (TIF) [file pone.0266347.s009.tif]

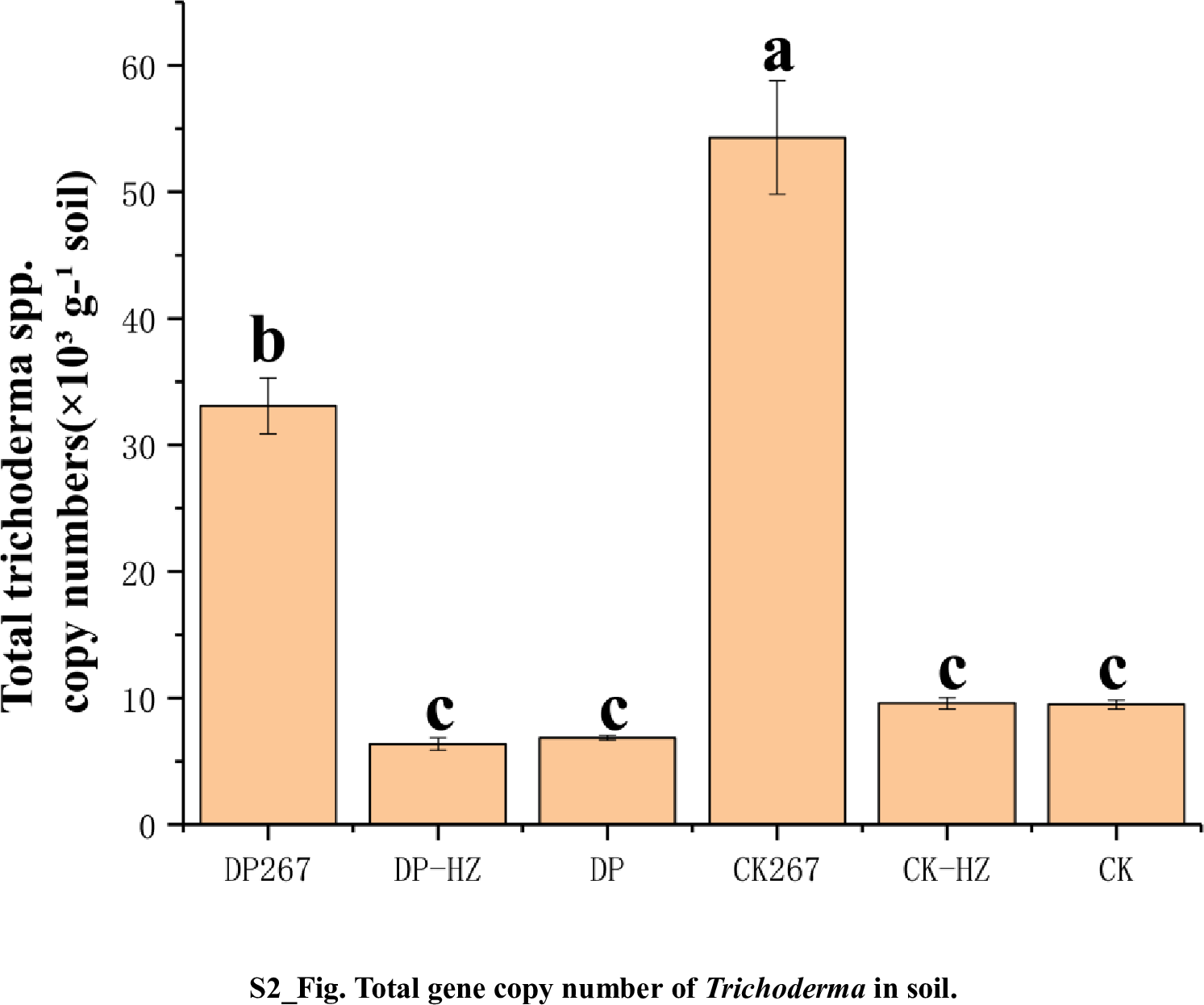

Supplement: S2 Fig — DP267 = Trichoderma strain 267 added after fumigation; DPHZ = Commercial T. harzianum added to soil after fumigation. CK267 = Trichoderma strain 267 added to soil without fumigation. CKHZ = Commercial T. harzianum added to soil without fumigation. DP = Fumigation without Trichoderma. CK = Untreated control. Means (N = 3) within the same time period accompanied by the same letter were not statistically different (P = 0.05), according to Duncan’s new Multiple-Range test. (TIF) [file pone.0266347.s010.tif]

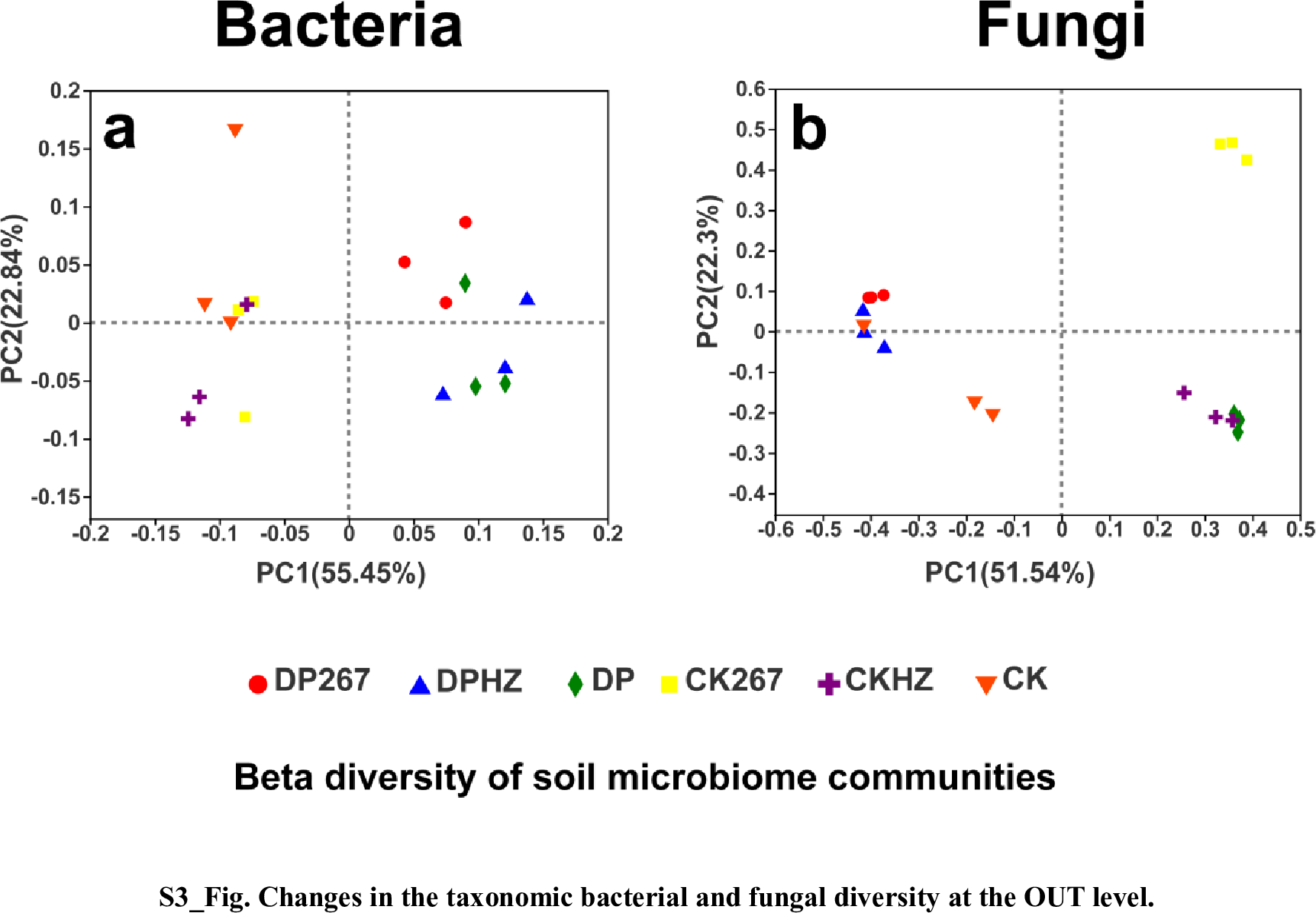

Supplement: S3 Fig — DP267 = Trichoderma strain 267 added after fumigation (see 2.2.2. in the text for detail); DPHZ = Commercial T. harzianum added to soil after fumigation. CK267 = Trichoderma strain 267 added to soil without fumigation. CKHZ = Commercial T. harzianum added to soil without fumigation. DP = Fumigation without Trichoderma. CK = Untreated control. (TIF) [file pone.0266347.s011.tif]

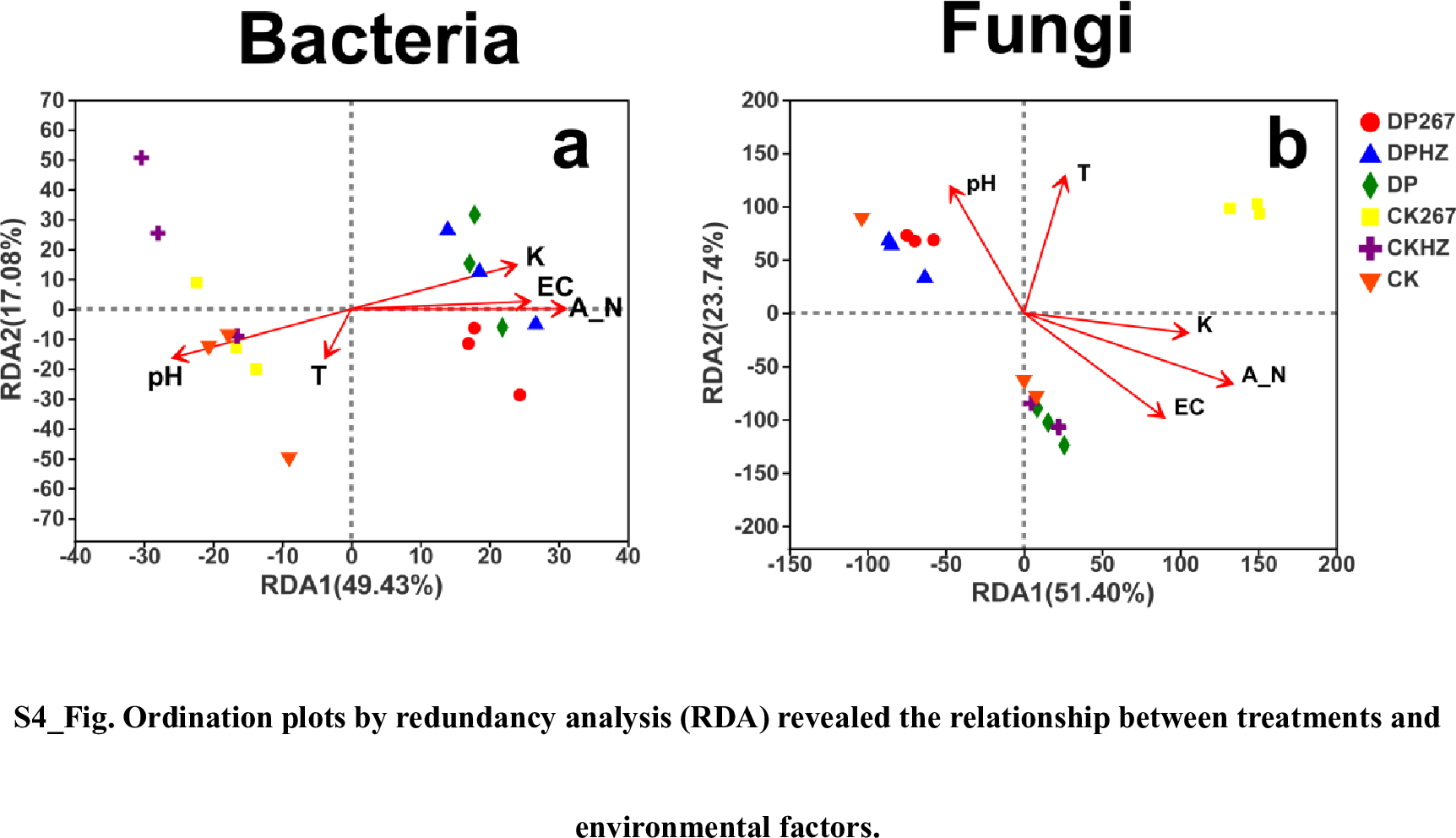

Supplement: S4 Fig — pH = pH of the soil; T = The relative abundance of Trichoderma in soil; K = Available potassium; A-N = Ammonium nitrogen; EC = Electrical conductivity. The treatments were: DP267 = Trichoderma strain 267 added to soil after fumigation. DPHZ = Commercial T. harzianum strain added to soil after fumigation. DP = Fumigation without the addition of Trichoderma. CK267 = Trichoderma strain 267 added to unfumigated soil. CKHZ = Commercial T. harzianum strain added to unfumigated soil. CK = Untreated control. (TIF) [file pone.0266347.s012.tif]
