# Supplementary material for: Beneficial effect on the soil microenvironment of Trichoderma applied after fumigation for cucumber production
Source: PLoS One. 2022 Aug 2;17(8):e0266347. doi: 10.1371/journal.pone.0266347 (PMC9345367; doi:10.1371/journal.pone.0266347)
Supplement: Attachment — Submitted filename: Mansucript_PONE-D-22-00468.docx [file pone.0266347.s014.docx]

This manuscript is generally well-written and the work was well-executed and informative. There are some points, which need to be clarified, which I have indicated directly in the edited version attached to the manuscripts.

The title, abstract and keywords are enough to present what was searched and the main results.

The introduction is well written and justifies what was done in the research.

Materials and methods are well written. However, some adjustments should be made for better understanding by the reader (suggestions in the comments in the document).

Overall, the discussion is well written and explains the results. However, some points should be detailed for better understanding by the reader (check in the comments)

Conclusions are ok.

**Suggestions**

**Page 6**

Line 13: Add air temperature and relative humidity

**Page 7**

Line 20: Add after how many days was germination

**Page 8**

Line 1 - 3: Detail the climatic conditions at the time of transplantation

Line 2: Add the volume of the pots

**Page 8**

Line 1: Detail how the pigments were determined

**Page 25**

Line 17 -20: Authors should explain why *Trichoderma* provided plant growth. In relation, the greater availability of nutrients and, consequently, greater possibility of plants to absorb forms of N and P. Chlorophyll contents should be explored

**Page 26**

Line 09 -11: Authors should explain why the nitrification of ammonia and denitrification of nitrate in soil is reported to change the relative abundance of NH4^+^ -N and NO3^-^N continuously

**Page 30**

Line 11 -13: Authors should describe in greater detail how the use of Trichoderma reduces the presence of nematodes
