# Supplementary material for: Beneficial effect on the soil microenvironment of Trichoderma applied after fumigation for cucumber production
Source: PLoS One. 2022 Aug 2;17(8):e0266347. doi: 10.1371/journal.pone.0266347 (PMC9345367; doi:10.1371/journal.pone.0266347)
Supplement: Attachment — Submitted filename: Response to Reviewers.docx [file pone.0266347.s015.docx]

Manuscript ID: PONE-D-22-00468

Title: "Beneficial effect on the soil microenvironment of *Trichoderma* applied after fumigation

for cucumber production "

Jiajia Wu, Jiahong Zhu, Daqi Zhang, Hongyan Cheng, Baoqiang Hao, Aocheng Cao, Dongdong Yan, Qiuxia Wang, Yuan Li

**Response Letter**

Dear Editor Rashid Nazir,

Thank you very much for your letter and suggestions. We have revised the manuscript. We would like to resubmit it for your consideration. We have addressed the comments raised by the reviewers. Point by point responses to the reviewers’ comments are listed below this letter. We hope that the revised manuscript is now acceptable for publication in your journal.

We look forward to hearing from you soon.

With best wishes,

Yours sincerely,

Yuan Li

We would like to express our sincere thanks to you and the reviewers for the careful and valuable comments on the manuscript.

**Replies to the reviewers:**

**Response to reviewer #2:**

1. Add air temperature and relative humidity.

Response:

Thank you for your comments. We have added air temperature and relative humidity according to your suggestion. Soil was fumigated with both DMDS and PIC (DP) in a greenhouse (day/night temperature 28℃/16℃; relative humidity 50−70%; day length 10 h) in Shunyi District, Beijing (40° 13′ N, 116° 65′ E). We have added this part in **line 5-6 on page 6** of Materials and methods of the manuscript.

2. Add after how many days was germination.

Response:

Thank you for your comments. We have added “how many days was germination” according to your suggestion. Cucumber seeds (Jingyou 4, Beijing Wanlongyufeng Seed Co., Ltd., China) were soaked in water at 60°C for 5 h, then placed on sterile wet filter paper in 150 mm diameter petri dishes at 28℃ and 95% humidity, after 1 day was germination. We have added this part in **line 16 on page 7** of Materials and methods of the manuscript.

3. Detail the climatic conditions at the time of transplantation.

Response:

Thank you for your comments. At 26℃ and 60% relative humidity, the cucumber seedlings were transplanted from the tray to individual pots (120 ×130 mm) when the seedlings were in the ‘three-leaf and heart’ stage. We have added this part in **line 15 on page 7** of Materials and methods of the manuscript.

4. Add the volume of the pots.

Response:

Thank you for your comments. At 26℃ and 60% humidity, the cucumber seedlings were transplanted from the tray to individual pots (120 ×130 mm) when the seedlings were in the ‘three-leaf and heart’ stage. We have added the volume of the pots in **line 18 on page 7** of Materials and methods of the manuscript.

5. Detail how the pigments were determined.

Response:

Thank you for your comments. A hand-held, non-destructive SPAD-502 Chlorophyll Meter (Konica Minolta, Inc., Japan) was used measure the amount of chlorophyll present in the cucumber plant leaves in each treatment. We have described this part in greater detail in **line 18-21 on page 8** of Materials and methods of the manuscript.

6. Authors should explain why *Trichoderma* provided plant growth. In relation, the greater availability of nutrients and, consequently, greater possibility of plants to absorb forms of N and P. Chlorophyll contents should be explored.

Response:

Thank you for your comments. *Trichoderma* can improve plant growth, which may be due to nutrient use efficiency and photosynthesis. Some *Trichoderma* strains have been found to enhance plant nutrient uptake and nitrogen use efficiency. Rudresh and colleagues have indicated that the application of *Trichoderma* enhance chickpea growth, nutrient uptake possibly due to an increased availability of nutrients and a release of growth promoting substances produced by the microbes. Zhou et al. reported that *T. harzianum* promoted the growth of cucumber seedlings by enhancing root growth and photosynthetic capacity. We have explained this part **on page 28-29** of Discussion of the manuscript.

7. Authors should explain why the nitrification of ammonia and denitrification of nitrate in soil is reported to change the relative abundance of NH4^+^ -N and NO3^-^N continuously.

Response:

Thank you for your comments. Typical nitrification refers to the process of oxidizing NH_3_ or NH_4_^+^ to nitrate via hydroxylamine and nitrite under aerobic conditions and the participation of nitrifying microorganisms (NH_3_/NH_4_^+^→NH_2_OH→NO_2_^−^→NO_3_^−^). Denitrification means that denitrifying microorganisms gradually reduce NO_3_^-^ and release N_2_ or N_2_O. Therefore, the nitrification of ammonia and denitrification of nitrate in soil to change the relative abundance of NH_4_^+^-N and NO_3_^-^-N continuously. We have explained this part in **line 13-20 on page 29** of Discussion of the manuscript.

8. Authors should describe in greater detail how the use of *Trichoderma* reduces the presence of nematodes.

Response:

Thank you for your comments. That may be due to the addition of *Trichoderma* can have direct and indirect effects on nematode, including inducing resistance in plant, and reducing nematode feeding and egg hatching. Yan et al. found that *T. harzianum* could enhance tomato resistance to root-knot nematode by stimulating secondary metabolism and defense enzyme activity that greatly contribute to alleviate oxidative stress and suppress root-knot nematode infections. We have described this part in greater detail in **line 15-21 on page 33** of Discussion of the manuscript.
